# Laparoscopic assisted pancreaticoduodenectomy: an important link in the process of transition from open to total laparoscopic pancreaticoduodenectomy

**DOI:** 10.1186/s12893-020-00752-5

**Published:** 2020-05-06

**Authors:** Feng Tian, Yi-zhi Wang, Su-rong Hua, Qiao-fei Liu, Jun-chao Guo

**Affiliations:** Department of General Surgery, Peking Union Medical College Hospital, Chinese Academy of Medical Sciences and Peking Union Medical College, No.1, Shuaifuyuan, Wangfujing Avenue, Dongcheng District, Beijing, 100730 China

**Keywords:** Open pancreaticoduodenectomy, Laparoscopic assisted pancreaticoduodenectomy, Pancreatic fistula, Safety

## Abstract

**Background:**

The safety of total laparoscopic pancreaticoduodenectomy still remains controversial. Laparoscopic assisted pancreaticoduodenectomy (LAPD) may be an alternative selection. The purpose of the present study is to compare a consecutive cohort of LAPD and open pancreaticoduodenectomy (OPD) from a single surgeon.

**Methods:**

A comparison was conducted between LAPD and OPD from January 2013 to December 2018. Perioperative outcomes and short-term oncological results were compared. Univariate and multivariable analyses were performed to determine associations among variables.

**Results:**

133 patients were enrolled, 36 patients (27.1%) underwent LAPD and 97 (72.9%) underwent OPD. No 30-day and 90-day mortality occurred. LAPD was associated with decreased intraoperative estimated blood loss (300 versus 500 ml; *P* = 0.002), longer operative time (372 versus 305 min; *P* < 0.001) compared with OPD. LAPD had a conversion rate of 16.7%, and wasn’t associated with an increased grade B/C pancreatic fistula rate, major surgical complications, intraoperative blood transfusion, reoperation rate or length of hospital stay after surgery. In the subset of 58 pancreatic ductal adenocarcinomas, R0 resection rate, median total harvested lymph node or lymph nodes ≥12 did not differ between the two groups.

**Conclusion:**

LAPD could be performed with non-inferior short-term perioperative and oncologic outcomes achieved by OPD in selected patients.

## Background

In recent years, minimally invasive pancreaticoduodenectomy (PD) are increasingly reported due to its potential benefits to patients since first described in 1994 by Gagner [1]. However, the wide spread of robotic PD is limited by its high cost, and total laparoscopic PD (TLPD) still restricts to a few high-volume pancreatic centers, owing to the required challenging technique. What’s more, the controversial safety remains the main concern during wide implementation [2]. A recent multi-institutional data from China reported that 30-day mortality and reoperation rates of minimally invasive PD were higher than that of matched OPD cases [3]. Also, two western studies reported increased complication-related mortality of TLPD compared with OPD [2, 4]. Safety metrics is paramount during the wide implementation of minimally invasive PD. It is necessary to find a minimally invasive approach suitable for learning and implementation.

Laparoscopic assisted pancreaticoduodenectomy (LAPD), a hybrid procedure combining laparoscopic resection and reconstruction under a small incision, may serve as an alternative on the road to matured application of TLPD. The potential advantages of LAPD include more precise mobilization and dissection compared with OPD, and more precise reconstruction and hemostasis compared with TLPD, which will possibly lead to a more favorable postoperative recovery. Limited literature described the safety and efficacy of LAPD. Meanwhile, comparative study lacked for LAPD and OPD [5]. The purpose of the present study was to compare the perioperative outcomes of LAPD and OPD in a large consecutive cohort of patients.

## Methods

### Study design and patient enrollment

**Fig. 1:**
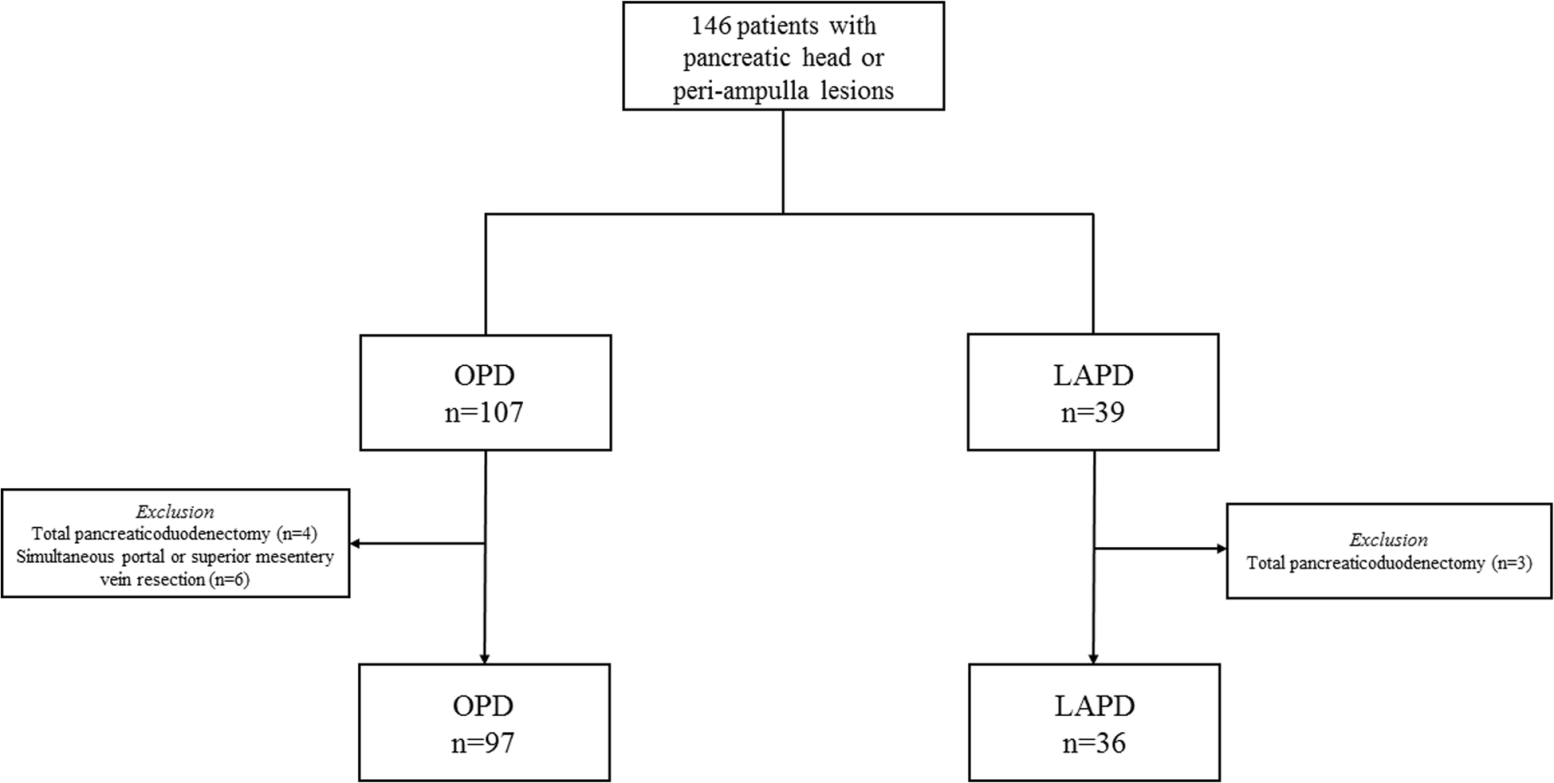
Study flow chart

Patients who underwent LAPD or OPD for resectable pancreatic head or peri-ampulla lesions from January 2013 to December 2018 were included in this retrospective study. LAPDs in this study started in March 2017. Intention-to-treat analysis was employed, such that LAPD cases that were converted to open were analyzed in LAPD group. Surgical indications referred to the National Comprehensive Cancer Network Guidelines for pancreatic tumors [6]. Cases with evidence of distant metastasis, requiring concomitant vascular resection, or multi-visceral resections (e.g., colon, total pancreas and spleen) due to adjacent organ involvement were excluded. Short-term oncologic outcomes were compared only among patients diagnosed pancreatic ductal adenocarcinoma (PDAC). All investigators were trained in standard criteria and procedures during data collection. This study was approved by the ethics committee of Peking Union Medical College Hospital. All patients signed written informed consent. The study flow chart was shown in Fig. 1.

### Main outcomes and definitions

The primary outcome was postoperative mortality (30-day and 90-day). Other safety-related variables including major postoperative complications (Clavien-Dindo classification grade III–V) [7], intraoperative estimated blood loss (EBL), conversion rate to open surgery, postoperative pancreatic fistula (POPF) rate (defined by the 2016 ISGPF criteria: Biochemical leak: postoperative day 3 drainage amylase > 3-times upper limit of normal value (0–135 U/ml in the authors’ institution), without clinical impact. Grade B fistula: clinical managements of POPF are needed due to fistula related infection or bleeding, such as percutaneous/endoscopic drainage, angiographic procedures or prolonged persistent drainage>3 weeks. Grade C fistula: POPF related complications needing reoperation, causing organ failure or even death) [8], and re-operative rate. Efficiency-related variables included operative time (OT, skin to skin time), time to first flatus, and postoperative length of stay (LOS). Histopathologic outcomes included final pathologic diagnosis, maximum tumor diameter, lymphovascular invasion, perineural invasion, R0 resection (defined as tumor within 1 mm of the resected margin), number of harvested lymph nodes.

Demographic information was also collected, including age, sex, body mass index (BMI), preoperative hemoglobin, albumin level, American Society of Anesthesiologists (ASA) classification, and symptoms. Definition of postoperative complications were reported in previous works [9–12].

### Surgical techniques and management

**Fig. 2:**
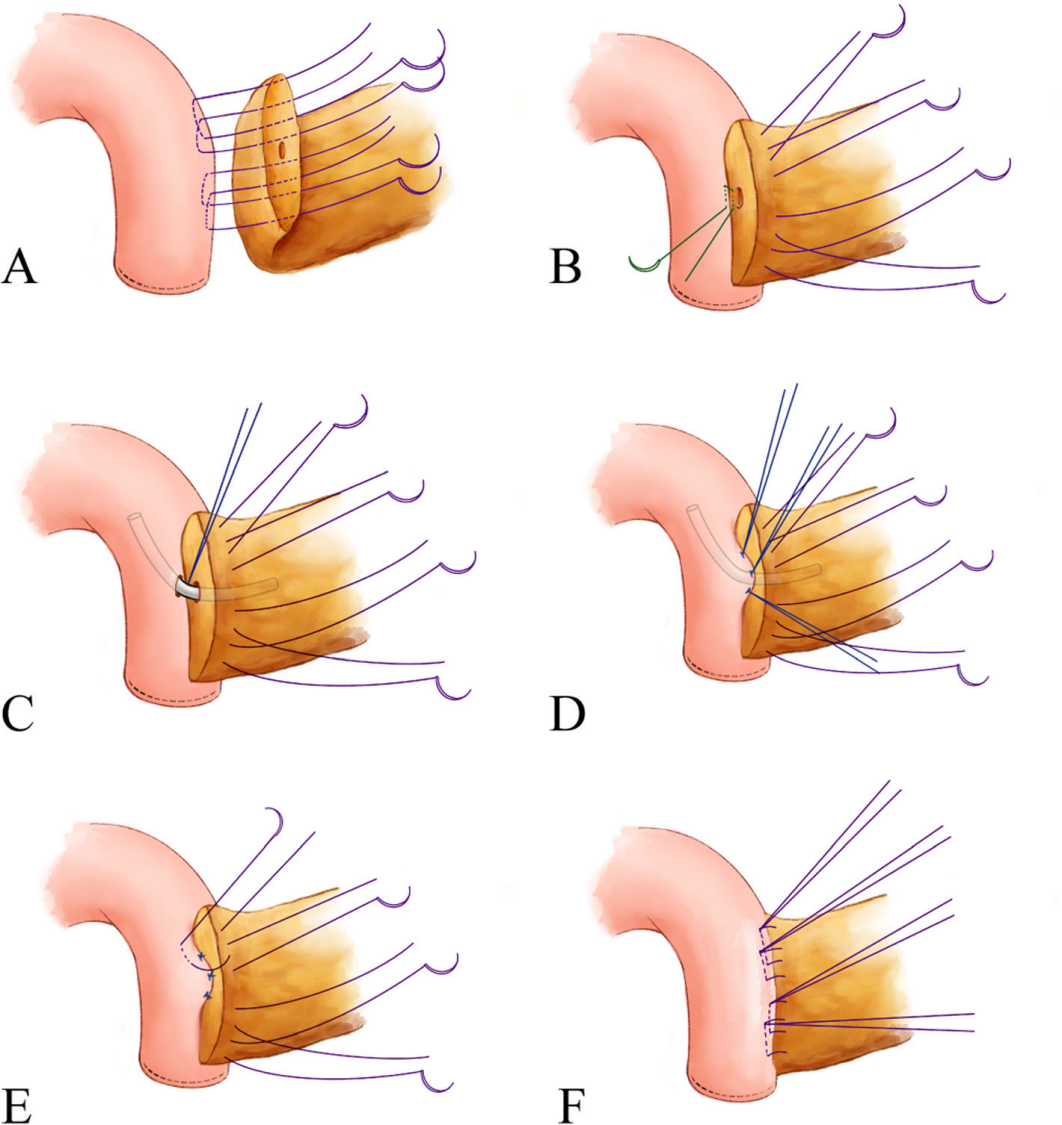
Steps of modified Blumgart technique in pancreatojejunostomy. Panel **a**, four to five “U” style stitches are placed superior and inferior the pancreatic duct, which passes through entire layer of the pancreatic stump and seromuscular layer of the jejunum (3–0 suture). Panel **b**, a separate horizontal interrupted stitch is then placed below the duct (4–0 suture). Panel **c**, a stent is routinely placed after making a small hole through the jejunal wall, which is fixed by the 4–0 suture. Panel **d**, four duct-to-mucus vertical interrupted stitches are subsequently placed at approximate 2, 4, 8, 10 o’clock, respectively (5–0 suture). Panel **e**, all 3–0 sutures are tightened and then pass through seromuscular layer of the anterior jejunum. Panel **f**, tie all the 3–0 sutures and a stable pancreatojejunostomy is finished

OPD was carried out through right upper transrectus incisions as low as two centimeters below the navel. A “Region Oriented” concept was commonly applied during resection portion of PD procedure in the authors’ institution. It contained four main regions, including infra-pancreatic region (to dissect the anterior and right wall of superior mesentery vein and ligate its branches in this area, including Henle’s trunk, upper right colic vein, veins from the pancreatic dorsal, and show the PV-SMV axis), lateral-duodenal region (to fully dissect this area using Kocher’ maneuver until showing the profile of superior mesentery artery), superior pancreatic region (to dissect the supera-pancreatic stereoscopic triangle consisting of common hepatic artery, gastroduodenal artery, superior border of the pancreas, and anterior wall of portal vein), and uncinated region (to dissect and ligate first jejunal vein, superior/inferior pancreaticoduodenal vein/artery). Lymph node harvest was integrated into these four steps. Principle of total mesopancreas excision (TMpE) was followed if malignant tumor was suspected. For reconstruction, modified Blumgart was used for pancreatojejunostomy as shown in Fig. 2, during which a pancreatic stent was routinely placed. Continuous suture with a 5–0 PDS (Ethicon, Somerville, NJ, USA) was adapted for hepaticojejunostomy, and a two-layer hand suture was routinely applied for gastrojejunostomy.

Regarding to LAPD, the resection portion was performed similarly with that of OPD, except the “superior mesentery artery first” procedure when dissecting the pancreatic uncinated process [13]. An approximate 10-cm (the size of one fist) right upper transrectus incision was then made for the subsequent reconstruction portion. Unreliable vascular branches were treated precisely through this incision before reconstruction if needed.

Prophylactic antibiotics were administered 30 min before skin incision. Two intraoperative peritoneal drains (one behind hepaticojejunostomy via the Winslow hole, another near pancreatojejunostomy) were routinely placed in all cases. Somatostatin analogues such as Octreotide (Merck Serono, Aubonne, Switzerland) were used for 3 days postoperatively.

### Statistical analysis

Statistical analysis was conducted using SPSS® version 23.0 (IBM Corporation, Armonk, NY, USA). Categorical variables were descripted as frequencies and percentages, and continuous variables were expressed as median (range) or mean (s.d.) after testing for normality. The univariate association of each covariate with the two surgical approaches (LAPD or OPD) was assessed using Mann–Whitney U test or Student’s t test for continuous variables, and categorical variables were analyzed by means of χ2 and Fisher’s exact test, where appropriate. Linear or logistic regression models were applied for adjusted analyses. Variables with *P* < 0.10 in univariate regression analyses were further evaluated in multivariate logistic regression models. Results of logistic regression were presented as odds ratios (ORs) plus 95% confidence intervals (CIs). *P* < 0.05 was considered statistically significant.

## Results

A total of 146 consecutive patients underwent PD procedures, of whom 107 (73.3%) had OPDs and 39 (26.7%) via LAPD approaches. Three patients in the LAPD group (total pancreaticoduodenectomy) and ten in the OPD group (6 underwent simultaneous portal or superior mesentery vein resection; 4 total pancreaticoduodenectomy) were excluded.

The characteristics of the enrolled 133 patients were depicted in Table 1. Median patient age was 59 years and 58.6% were male. Median OT and EBL were 320 min and 400 ml, respectively, with 27.1% of cases requiring intraoperative blood transfusion and 21.1% undergoing pylorus preservation PD. PD was performed for PDAC in 43.6% of cases (58 patients), peri-ampulla malignancies in 33.8% (45 patients), other non-PDAC pancreatic lesions in 20.3% (27 patients, mainly including solid pseudopapillary tumor, intraductal papillary mucinous neoplasm, neuroendocrine tumor, and cystic tumor), and chronic pancreatitis in 2.3% (3 patients).

**Table 1:** Comparison of clinicopathological characteristics and perioperative outcomes between OPD and LAPD groups

Variable	All PDs	OPD(***n*** = 97)	LAPD(***n*** = 36)	***P***
Age, median (range), yrs	59 (14–79)	58 (14–79)	60.5 (17–78)	0.147
Male sex	78 (58.6)	59 (60.8)	19 (52.8)	0.402
ASA ratio (I: II: III)	18:103:12	16:70:11	2:33:1	0.068
BMI, mean (SD), kg/m^2^	22.26 (2.87)	22.5 (2.9)	21.8 (2.7)	0.211
Abdominal/Back Pain	70 (52.6)	55 (56.7)	15 (41.7)	0.123
Jaundice	60 (45.1)	45 (46.4)	15 (41.5)	0.627
Preoperative ALB	40.4 (6.7)	40.7 (4.4)	41.8 (4.7)	0.234
Preoperative HGB	127.3 (19.7)	128.3 (16.6)	127.9 (15.7)	0.895
Lesion major diameter, median (range), cm	3.0 (0.2–12.5)	3.0 (0.2–12.5)	2.65 (0.4–6.5)	0.594
PDAC	58 (43.6)	47 (48.5)	11 (30.6)	0.064
Pylorus-preserving PD	28 (21.1)	17 (17.5)	11 (30.6)	0.101
OT, median (range), min	320 (210–585)	305 (210–585)	372 (257–525)	< 0.001
EBL, median (range), ml	400 (50–2400)	500 (50–2400)	300 (50–1300)	0.002
Intraoperative blood transfusion	36 (27.1)	30 (30.9)	6 (16.7)	0.100
Conversion to open	–	–	6 (16.7%)	–
30-Day mortality	0	0	0	–
90-Day mortality	0	0	0	–
Grade B/C POPF	1 (0.8)	1 (1.0)	0 (0)	1.000
DGE	30 (22.6)	22 (22.7)	8 (22.2)	0.955
Major complication (Grade III-V)	40 (30.1)	30 (30.9)	10 (27.8)	0.725
Grade I	8 (6.0)	6 (6.2)	2 (5.6)	1.000
Grade II	27 (20.3)	20 (20.6)	7 (7.3)	0.881
Grade III	37 (27.8)	28 (28.9)	9 (25.0)	0.658
Grade IV	3 (2.3)	2 (2.1)	1 (2.8)	1.000
Grade V	0 (0)	0 (0)	0 (0)	–
Time to first flatus, median (range), days	4 (1–9)	4 (1–9)	3 (2–8)	0.007
Re-operation	5 (3.8)	4 (4.1)	1 (2.8)	1.000
Postoperative LOS, median (range), days	12 (7–51)	12 (7–51)	11.5 (7–35)	0.472

Values in parentheses are percentages unless indicated otherwise; **Abbreviations**: *ALB* Albumin, *ASA* American Society of Anesthesiologists, *BMI* body mass index, *DGE* delayed gastric emptying, *OT* operative time, *EBL* estimated blood loss, *HGB* Hemoglobin, *LAPD* laparoscopic assisted pancreaticoduodenectomy, *LOS* length of hospital stay, *OPD* open pancreaticoduodenectomy, *PD* pancreaticoduodenectomy, *PDAC* Pancreatic ductal adenocarcinoma, *POPF* postoperative pancreatic fistula

LAPD and OPD patients did not differ in baseline data (age, sex, BMI), clinical features (symptoms, ASA classification, state of nutrition, maximum tumor size), PDAC proportion, or pylorus-preservation rate (all *P*>0.05).

### Perioperative and short-term oncologic outcomes

The perioperative outcomes of the study were displayed in Table 1. No 30-day and 90-day mortality occurred in both groups. As compared with OPD, LAPD was associated with longer OT (median 372 versus 305 min; *P* < 0.001), lower EBL (median 300 versus 500 ml; *P* = 0.002), shorter time to first flatus (median 3 versus 4 days; *P* = 0.007). Six (16.7%) patients in the LAPD group required conversion to open surgeries because of failure to progress or hemorrhage. Intraoperative blood transfusion tended to occur more frequently in the OPD group than in the LAPD group (30.9% versus 16.7%), but no significance was detected (*P* = 0.100). The overall rate of grade B/C POPF was 0.8% (1 out of 133), with 1.0% and zero in the OPD and LAPD group, respectively (*P* = 1.000). Major complication rates (27.8% versus 30.9%), rate of decreased hemoglobin and albumin, re-operative rate, and postoperative LOS were statistically similar between the groups (all *P*>0.05).

Short-term oncologic outcomes of the 58 (43.6%) PDAC cases were analyzed separately (Table 2). R0 resection rate were 90.9 and 63.8% in the LAPD and OPD groups, respectively (*P* = 0.166). Both groups underwent high quality of lymph node harvest, with similar total harvested lymph nodes. Specifically, 100.0% of cases in the LAPD group obtained no less than 12 lymph nodes.

**Table 2:** Surgical quality for 58 PDACs and comparison between the two groups

Variable	All PDs	OPD(***n*** = 47)	LAPD(***n*** = 11)	***P***
R0 resection	40 (69.0)	30 (63.8)	10 (90.9)	0.166
Harvested lymph nodes, median (range)	21 (4–56)	21 (4–56)	21 (12–38)	0.456
≥12 Lymph nodes harvested	55 (94.8)	44 (93.6)	11 (100.0)	1.000
Positive lymph nodes, median (range)	1 (0–9)	1 (0–9)	0 (0–7)	0.479

Values in parentheses are percentages unless indicated otherwise; **Abbreviations**: *LAPD* Laparoscopic assisted pancreaticoduodenectomy, *OPD* Open Pancreaticoduodenectomy, *PD* Pancreaticoduodenectomy, *PDAC* pancreatic ductal adenocarcinoma

### Univariate and multivariate analyses of factors affecting main perioperative variables

Tables 3 and 4 displayed the univariate and multivariate analysis (MVA) of factors associated with EBL and OT, respectively. After adjusting for confounders, abdominal/back pain, typical Whipple procedure, and an increasing OT were significantly associated with increased EBL, whereas LAPD approach was associated with reduced EBL [*OR* = 6.720, *95% CI* 2.123–21.152]. On MVA, LAPD was confirmed to be independently associated with a reduction in EBL, whereas increasing OT was independently associated with increased EBL.

**Table 3:** Univariate and multivariate analyses of factors associated with EBL

Variables	OR	Univariate analysis	***P***	OR	Multivariate analysis	***P***
		95%CI			95%CI	
Male	1.895	0.930 3.859	0.078	1.503	0.675 3.343	0.318
Abdominal/back pain	0.446	0.221 0.901	0.024	0.651	0.293 1.445	0.291
Typical Whipple	0.353	0.138 0.901	0.029	0.717	0.237 2.173	0.557
Increasing OT	1.007	1.002 1.013	0.008	1.012	1.005 1.020	0.001
PDAC	0.556	0.277 1.115	0.098	0.742	0.321 1.716	0.486
LAPD	3.062	1.305 7.186	0.002	6.720	2.123 21.152	0.001

**Abbreviations**: *CI* confidence interval, *EBL* estimated blood loss, *LAPD* Laparoscopic assisted pancreaticoduodenectomy, *OR* odds ratio, *OT* operating time, *PDAC* pancreatic ductal adenocarcinoma

**Table 4:** Univariate and multivariate analyses of factors associated with OT

Variables	OR	Univariate analysis	***P***	OR	Multivariate analysis	***P***
		95%CI			95%CI	
Jaundice	0.527	0.264 1.053	0.070	0.481	0.211 1.098	0.082
Lose Body weight	0.536	0.263 1.093	0.086	0.516	0.215 1.239	0.139
ASA						
ASA I	3.717	0.951 14.530	0.059	2.988	0.711 12.551	0.135
ASA II	1.500	0.293 7.681	0.055	2.309	0.395 13.499	0.353
ASA III	Ref	Ref Ref	Ref	Ref	Ref Ref	Ref
LAPD	0.118	0.045 0.311	< 0.001	0.103	0.036 0.293	< 0.001

**Abbreviations**: *ASA* American Society of Anesthesiologists, *CI* confidence interval, *OR* odds ratio, *OT* operating time, *Ref* reference variable

Factors associated with major complications were listed in Table 5, slower first flatus, bigger tumor size, and more harvested lymph node (LN) were or tended to be associated with increased major complication rate on univariate analysis, whereas only slower first flatus was independently associated with increased major complications on MVA. Table 6 revealed results for factors associated with LOS. Growing age, cholangitis, intra-abdominal infection, and delayed gastric emptying (DGE) were independently associated with prolonged LOS. The surgical approach to PD was not predictive of LOS.

**Table 5:** Univariate and multivariate analyses of factors associated with major complication rate

Variables	OR	Univariate analysis	***P***	OR	Multivariate analysis	***P***
		95%CI			95%CI	
ASA						
ASA I	0.355	0.106 1.196	0.095	0.385	0.107 1.381	0.143
ASA II	0.636	0.145 2.784	0.548	0.320	0.064 1.605	0.166
ASA III	Ref	Ref Ref	Ref	Ref	Ref Ref	Ref
Bigger tumor size	−0.649^a^	−1.313 0.015	0.055	1.179	0.940 1.479	0.153
More harvested LN	2.418	1.000 5.850	0.050	0.961	0.919 1.005	0.078
Slower first flatus	−0.747^a^	−1.331 -0.163	0.013	1.389	1.072 1.799	0.013

^a^Values indicate mean difference; **Abbreviations**: *ASA* American society of anesthesiologists, *CI* confidence interval, *LN* lymph node, *OR* odds ratio, *Ref* reference variable

**Table 6:** Univariate and multivariate analyses of factors associated with postoperative LOS

Variables	OR	Univariate analysis	***P***	OR	Multivariate analysis	***P***
		95%CI			95%CI	
Increasing age	−4.987^a^	−9.464 -0.511	0.029	1.053	1.008 1.099	0.019
Reflux cholangitis	0.156	0.033 0.742	0.020	0.150	0.027 0.843	0.031
Abdominal infection	0.077	0.010 0.619	0.016	0.039	0.004 0.336	0.003
DGE	0.060	0.017 0.210	< 0.001	0.050	0.013 0.191	< 0.001
Slower first flatus	−0.722^a^	−1.257 -0.187	0.009	1.231	0.912 1.663	0.175

^a^Values indicate mean difference; **Abbreviations**: *CI* confidence interval, *DGE* delayed gastric emptying, *LOS* length of hospital stay, *OR* odds ratio

## Discussion

In the present study, we represented a large cohort of PDs via open or laparoscopic assisted approaches, which revealed that LAPD had non-inferior perioperative and short-term oncologic outcomes compared with that achieved in OPD.

As the most important safety outcome, no mortality occurred in the whole cohort, which was lower than the 2.43% reported by Wang et al. [3] and 2.6% by Zureikat et al. [14]. This reflected the high quality and safety of PD procedures in the present study, which might be contributed by relatively low rates of grade B/C POPF (0.8%), postoperative hemorrhage (2.3%) and re-operation (3.8%). POPF was the key point focused in the most published studies, because a failed pancreatic anastomosis could lead to serious bleeding, sepsis, and even death. Reported grade B/C POPF rate of PD via minimally invasive approaches varied between 6.51 and 12% among different countries [3, 14–19]. Regardless of surgeon’s experiences, pancreatojejunostomy achieved via laparoscopic approach seemly could not compete with manual work, owing to the difficulty of accurate needle handling under laparoscope. Pancreatic stent and duct-to-mucosa modified Blumgart were applied routinely in the authors’ pancreatojejunostomies, which combined with precise mobilization and dissection, resulted in a much lower clinically relevant POPF rate compared with previously reported data. Wang et al. also reported an 8.55% of re-operation rate, which was largely due to the notable POPF rate as demonstrated in the multivariate analysis [3]. Moreover, the postoperative hemorrhage rate of the present study compared favorably with that of 12.44% for 1089 laparoscopic cases from China [3]. One reason contributing to this included the chance offered by the assisted incision of LAPD to easily check and consolidate the unconvincing vessel branches.

The conversion rate of 16.7% in this early experience of LAPD was higher than 2.3–9.0% published recently [3, 14, 17, 19, 20], but lower than 30.0% reported early by Adam et al. [2]. The heterogeneity of conversion rate depends on learning curve, hospital volume, surgeons’ experiences, and especially patient selection [21]. Univariate and multivariable analyses demonstrated that LAPD approach was associated with less intraoperative EBL (300 versus 500 ml in OPD group, *P* = 0.002) and a tendency to a lower transfusion rate (16.7 versus 30.9% in OPD group, *P* = 0.100). The reduced EBL in the LAPD group was consistent with most data of minimally invasive PD reported in prior literature [3, 19, 22]. The improved visualization and reduced disruption to tissues might result in an improved hemostasis in LAPD group. This, however, might come at the expense of longer OT. Multivariable analysis also found LAPD was an independent factor of increased OT.

Notable major complication rates were observed in both groups, which were consistent with the results of recently published studies by Choi et al and Shi et al. [23, 24]. In detail, DGE was the main complication detected, which was proven to be one of risk factors for longer LOS [*OR* = 0.050, *95% CI* 0.013–0.191]. However, multivariate analysis did not find any potential risk factor of DGE. A study including 10,249 patients indicated that age elder than 65 years, male sex, BMI over 30 kg/m^2^, ASA classification no less than grade III, pylorus preservation, and longer OT could be unfavorable risk factors of DGE while preoperative chemotherapy was associated with decreased risk of DGE [25]. The reason for the difference might lie in the small sample size of our study, which could cause potential bias.

Multivariate analysis revealed that time to first flatus was independently associated with major complication rate [*OR* = 1.389, *95% CI* 1.072–1.799] whereas LAPD approach was associated with faster flatus (median 3 versus 4 days in OPD group, *P* = 0.007). The reduced blood loss and the relatively faster first flatus might have been translated into a reduced postoperative LOS in the LAPD group, but there was no difference in LOS between the two groups. The median LOS of the cohort was 12 days, which was comparable with findings reported in prior literature [3, 26, 27]. Multivariate analysis found that reasons of prolonged LOS were multifactorial. Besides DGE, increased age [*OR* = 1.053, *95% CI* 1.008–1.099], postoperative abdominal infection [*OR* = 0.039, *95% CI* 0.004–0.336], and reflux cholangitis [*OR* = 0.15, *95% CI* 0.027–0.843] were also independent risk factors for longer LOS.

The quality of surgical resection was crucial for improving the survival rate of PDAC patients. PD with total mesopancreas excision (TMpE) was reported to be associated with decreased EBL, increased number of harvested lymph nodes and negative resection margins rates. However, evidences of improved survivals in PDAC patients with TMpE were limited [28]. Wu et al. retrospectively analyzed 120 patients with pancreatic head cancer who received PDs with TMpE and revealed a 71.6% of R0 resection [29]. Besides, a recent laparoscope-related study reported that R0 resection rate and number of harvested lymph nodes reached 86% and 26, respectively, regardless of surgical procedures [30]. In the present study, all PD procedures for PDACs have abided by the TMpE principle. LAPD group achieved a tendency to higher R0 resection rate (90.9 versus 63.8% in OPD group, *P* = 0.166) and similar number of harvested lymph nodes, as compared with its open counterpart. Although the R0 rate of 69% in the whole cohort was lower than aforementioned studies, the R0 rate in LAPD group was competitive and supported the convincing surgical quality in LAPD resection portion.

This study had several limitations. First, potential selection bias could not be avoided without propensity score matching, though no significant differences in the baseline characteristics of both groups were detected. Second, the present study mainly focused on short-term outcomes, results of long-term follow-up are awaited to determine recurrence and survival rates.

## Conclusions

In conclusion, this study demonstrated that LAPD could achieve non-inferior outcomes in safety and short-term oncologic efficacy compared with OPD. LAPD could be recommended as an alternative bridge in the process of switching from open to total laparoscopic approach for PD.

## Data Availability

The original data and materials are available from the corresponding author on reasonable request.

## Abbreviations

ASA: American society of anesthesiologists
BMI: Body mass index;
CI: Confidence interval
DGE: Delayed gastric emptying
EBL: Estimated blood loss
LAPD: Laparoscopic assisted pancreaticoduodenectomy
LN: Lymph node
LOS: Length of stay
OPD: Open pancreaticoduodenectomy
OR: Odds ratio
OT: Operative time
PD: Pancreaticoduodenectomy
PDAC: Pancreatic ductal adenocarcinoma
POPF: Postoperative pancreatic fistula
TLPD: Total laparoscopic pancreaticoduodenectomy
TMpE: Total mesopancreas excision.
